# Application of high-performance magnetic nanobeads to biological sensing devices

**DOI:** 10.1007/s00216-018-1548-y

**Published:** 2019-01-09

**Authors:** Yasuaki Kabe, Satoshi Sakamoto, Mamoru Hatakeyama, Yuki Yamaguchi, Makoto Suematsu, Makoto Itonaga, Hiroshi Handa

**Affiliations:** 1 Department of Biochemistry, Keio University School of Medicine, 35 Shinnanomachi, Shinjuku-ku, Tokyo, 160-8582 Japan; 2 Japan Agency for Medical Research and Development, Core Research for Evolutional Science and Technology, Tokyo, 200-0004 Japan; 3 School of Life Science and Technology, Tokyo Institute of Technology, 4259 Nagatsuta-cho, Midori-ku, Yokohama, Kanagawa 226-8501 Japan; 4 FG Beads Development Section, Biotronics Laboratory, Tamagawa Seiki Co. Ltd, Ohyasumi, Iida, Nagano, 395-8515 Japan; 5 Healthcare Business Division, JVCKENWOOD Corporation, 3-12 Moriya-cho, Kanagawa-ku, Yokohama, Kanagawa 221-0022 Japan; 6 Department of Nanoparticle Translational Research, Tokyo Medical University, 6-2-2 Nishishinjuku, Shinjuku-ku, Tokyo, 160-0023 Japan

**Keywords:** Magnetic nanobeads, Biosensor, Immunoassay, Exosome, Liquid biopsy

## Abstract

Nanomaterials have extensive applications in the life sciences and in clinical diagnosis. We have developed magnetic nanoparticles with high dispersibility and extremely low nonspecific binding to biomolecules and have demonstrated their application in chemical biology (e.g., for the screening of drug receptor proteins). Recently, the excellent properties of nanobeads have made possible the development of novel rapid immunoassay systems and high-precision technologies for exosome detection. For immunoassays, we developed a technology to encapsulate a fluorescent substance in magnetic nanobeads. The fluorescent nanobeads allow the rapid detection of a specific antigen in solution or in tissue specimens. Exosomes, which are released into the blood, are expected to become markers for several diseases, including cancer, but techniques for measuring the absolute quantity of exosomes in biological fluids are lacking. By integrating magnetic nanobead technology with an optical disc system, we developed a novel method for precisely quantifying exosomes in human serum with high sensitivity and high linearity without requiring enrichment procedures. This review focuses on the properties of our magnetic nanobeads, the development of novel biosensors using these nanobeads, and their broad practical applications.

**Figure:**
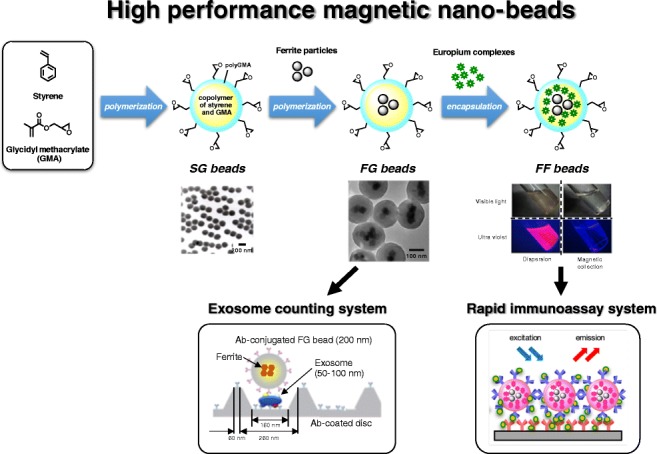
Graphical abstract

Graphical abstract

## Introduction

Magnetic materials have been used as magnetic storage systems, sensors, and shielding systems. Ferrite particles are frequently used as magnetic materials in the life sciences owing to their biocompatibility [1]. For instance, ferrite particles have been used as carriers for hyperthermia treatment and as MRI contrast agents coated with biocompatible materials, such as dextran [2]. Furthermore, they are widely used for the separation of bioactive substances (e.g., proteins, enzymes, and nucleic acids), blood screening, cell separation, and immunoassays [3, 4]. For these applications, magnetic materials should be subjected to coating and conjugation with appropriate materials on the surfaces of particles [5, 6]. With use of functionalized magnetic particles, a broad range of biosensing systems (e.g., immunoassays) for bioimaging or drug delivery have been developed [7–9]. Several types of magnetic particles have been developed for various biotechnological applications [10, 11]. For effective biosensor application, low nonspecific adsorptivity, high dispersibility, and high stability of magnetic particles are most important. We have focused on the development of high-performance nanoparticles for analyses in chemical biology, such as drug receptor protein screening [12]. Furthermore, we have recently demonstrated their applications as biosensors, for example, in novel immunofluorescent assays or exosome detection systems, utilizing the excellent properties of our magnetic nanobeads [13, 14]. This review describes the preparation and modification of nanobeads and their application in immunoassays.

## Development of high-performance magnetic affinity nanobeads

**Fig. 1:**
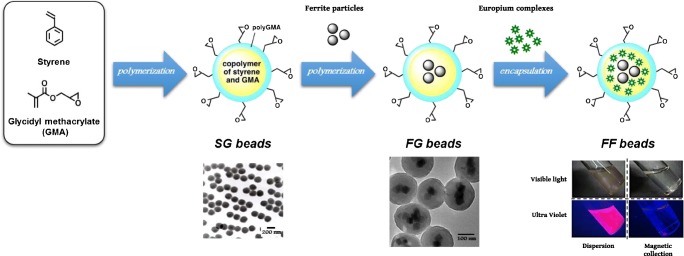
Construction of high-performance nanobeads. Flowchart of the construction of functionalized nanoparticles. SG beads are prepared by polymerization with styrene and glycidyl methacrylate (GMA) (left). FG beads are prepared with surface-modified ferrite particles, styrene, and GMA (middle). After ferrite particles have been covered by polymerization with styrene and GMA, the polymer-coated ferrite particles are further coated with GMA. Fluorescent FG beads (FF beads) are prepared from FG beads by incorporation with fluorescent molecules (europium complexes) in an organic solvent (right). Transmission electron microscopy images of SG beads and FG beads are shown. FF beads emit red fluorescence under ultraviolet light in solution. By the magnetic collection of FF beads, red fluorescence is collected at the bottom of the vial

Various types of chromatography, such as ion-exchange or gel-filtration chromatography, have been used for protein purification. Affinity chromatography is an effective technique, but efficient purification is limited by the nonspecific binding of proteins, insufficient chemical stability, and physical properties, such as dispersibility. We initially developed affinity latex beads (SG beads) as a carrier for affinity chromatography [15]. As shown in Fig. 1 (left), SG beads have polystyrene as a core and poly (glycidyl methacrylate) (polyGMA) on the surface, and their particle diameter is approximately 200 nm. SG beads have several beneficial characteristics, including (1) low nonspecific adsorption owing to their moderately hydrophilic and nonporous surface, (2) high dispersibility and mobility in various solvents owing to their stability, and (3) the potential to covalently immobilize several ligands, such as proteins, nucleic acids, or low molecular weight compounds, via epoxy groups derived from polyGMA on the surface. Therefore, SG beads allow the efficient purification of target proteins from crude extracts, such as tissues or cell lysates, with high recovery and purity. These excellent properties of SG beads have been used to successfully purify and identify several target proteins, such as transcription factors targeting specific DNA sequences and drug receptors [15–19].

To develop an automated system for affinity purification, we further developed highly functionalized magnetic beads (FG beads) with characteristics superior to those of SG beads. We found that compounds containing carboxyl or thiol groups strongly bind to magnetic iron oxide (ferrite); therefore, we used these compounds as “adapter molecules” for the modification of the ferrite surface. We prepared ferrite particles with diameters of 35–40 nm and coupled them to adapter molecules [20, 21]. The particles were coated with copolymer of styrene and GMA, and then the core beads were further coated by seed polymerization with GMA [20]. We confirmed that the FG beads were polymer microspheres encapsulating several ferrite nanoparticles with a diameter of approximately 200 nm by transmission electron microscopy (Fig. 1, middle). FG beads were further modified by introducing a spacer molecle ethylene glycol diglycidyl ether on the surface of beads (FGNEGDE). FGNEGDE beads exhibited high dispersion in various organic solvents, such as *N*,*N*-dimethylformamide, tetrahydrofuran, ethyl acetate, 1,4-dioxane, toluene, and dichloromethane, without the structural destruction of beads. Thus, FG beads have magnetic properties in addition to superior stability in organic solvents compared with SG beads. Using FG beads, we have identified target proteins for several low molecular weight compounds, including drugs [22, 23], toxins [24–26], metabolites (amino acids or heme) [27–29], and natural products [30]. Notably, FG beads allowed us to identify the target protein for thalidomide as cereblon, providing insight into the mechanism of action of thalidomide in teratogenicity and its anticancer effect against multiple myeloma [31–33]. Thus, FG beads could be a powerful tool for the identification of unknown target proteins for several ligands. In addition, we developed an automated system for affinity purification based on magnetic separation using FG beads. This system allows the systematic identification of target proteins by the simultaneous analysis of multiple samples [12]. FG beads and the automated screening equipment are commercially available from Tamagawa Seiki Co.,Ltd. (http://www.magneticnanoparticle.jp/jp/htdocs/index.html).

## Development of fluorescence-encapsulated magnetic nanobeads

In addition to their use for affinity purification, we applied FG beads in the development of novel biosensors. Biomarkers in biological fluids provide important information regarding disease progression and prognosis [34–37]. Various types of bioassays, including immunoassays, are used for measurement of biomarkers [38, 39], but these assays are often time-consuming and insufficient to obtain reliable results. For instance, although enzyme-linked immunosorbent assay (ELISA) is widely used as a standard diagnostic test, the completion of the reaction takes a long time [40, 41]. Various approaches have been evaluated to resolve these intrinsic issues. In the biomedical research field, appropriate functional materials or particles allow efficient diagnosis and treatment [42]. Magnetic particles are frequently used as efficient and sensitive functional materials [43, 44]. We developed a novel type of fluorescence-encapsulated magnetic nanobead for immunoassays.

To prepare fluorescent particles, fluorescent substances are generally immobilized on the surface of polymer particles by covalent or affinity binding, such as biotin–avidin coupling [45]. However, in this case, there were several issues, such as impaired dispersibility of the particles or increased nonspecific adsorption because the surface was covered with fluorescent molecules. Furthermore, it was difficult to functionalize the fluorescent particles because of a decrease in the number of surface functional groups. Ando and Kawaguchi [46] synthesized fluorescent particles by adding a fluorescent substance to a monomer solution before polymerization. The fluorescent particles were able to immobilize proteins electrostatically, but it was difficult to include functional groups on the surface of particles because the fluorescent substance leaked from the inside of particles in organic, acidic, or alkaline solutions. A method to enclose fluorescent molecules in polymer particles by regulating environmental conditions in solution has recently been evaluated [47]. Some polymer structures exhibit expansion and shrinkage depending on the environmental parameters, such as temperature, pH, light, or solvent exchange [48]. Therefore, we attempted to incorporate fluorescent substances into FG beads by altering the environmental conditions in the solution.

FG beads are tolerant to several organic solvents; however, we found that the FG bead expands or shrinks according to the type of solvent. In particular, the polymer structure of FG beads swells in acetone and returns to its original configuration in water. The fluorescent substances yttrium (Y), cerium (Ce), europium (Eu), and terbium (Tb), which are rare earth elements, each have peculiar fluorescence wavelengths and can control excitation wavelengths by forming chelate complexes. Eu has been used for labeling with nucleic acids or antibodies [49]. We used a fluorescent Eu derivative, tris(4,4,4-trifluoro-1-(2-thienyl)-1,3-butanediono) europium (Eu(TTA)_3_(TOPO)_2_), for incorporation into FG beads. Eu(TTA)_3_(TOPO)_2_ was mixed with FG beads in an acetone solution for swelling, and the solvent was removed by quick vacuum drying and added to an aqueous solution for shrinking. In this way, we successfully incorporated Eu(TTA)_3_(TOPO)_2_ into the polymer layer of FG beads [14] (Fig. 1, right).

The fluorescent FG beads (FF beads) emitted a strong red-orange color under ultraviolet light; when the beads were collected with a magnet, the fluorescence was drawn to the magnet (Fig. 1, right). FF beads emit fluorescent light at 618 nm with excitation at 340 nm. Additionally, FF beads maintained good dispersibility in aqueous solution. The fluorescence intensity of Eu(TTA)_3_(TOPO)_2_ is shifted by changes in conditions, such as the pH and buffer, and is suppressed by the addition of quenchers, such as ethylenediamine tetraacetic acid (EDTA). However, the dispersion of FF beads in micellar solution with various pH values and EDTA did not affect the fluorescence intensity of FF beads [50]. There was little nonspecific adsorption of the protein by FF beads owing to polyGMA on the surface. FF beads contained approximately 4.5 × 10^5^ molecules of Eu(TTA)_3_(TOPO)_2_ in a single particle, as determined by inductivity coupled plasma optical emission spectroscopy, and this enabled us to observe a single particle using a normal fluorescence microscope. In addition, FF beads did not cause density extinction, as is typical of fluorescent molecules. These findings indicated that FF beads can be used to detect time-resolved fluorescence with high sensitivity and low background noise. Furthermore, several ligands can be conjugated to the surface of the beads because the surface is not covered with fluorescent molecules. On the basis of these properties, we developed an immunoassay using FF beads.

## Development of a novel rapid immunoassay using FF beads

**Fig. 2:**
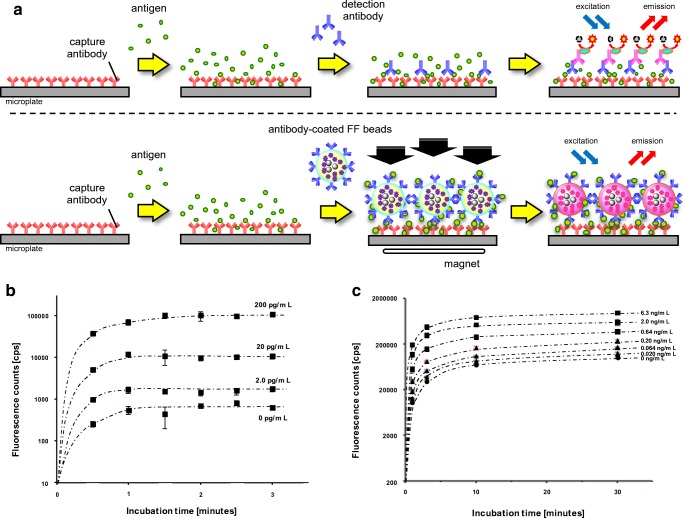

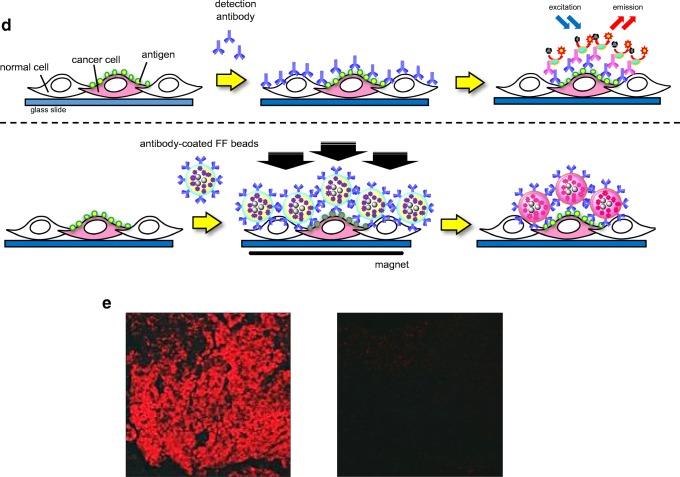
Development of a rapid immunoassay system using magnetic nanobeads. **a** A standard sandwich immunoassay (top) and a magnetically promoted sandwich immunoassay using antibody-conjugated fluorescent FG beads (FF beads) (bottom). **b** Detection of brain natriuretic peptide (BNP) by the magnetically promoted sandwich immunoassay using anti-BNP-conjugated FF beads. The graph shows the fluorescence intensity for the detection signal in the presence of BNP at concentrations of 0, 2.0, 20, and 200 pg/mL at the indicated time. All data are presented as the mean ± the standard deviation (*n* = 4). **c** Detection of prostate-specific antigen (PSA) by the magnetically promoted sandwich immunoassay using anti-PSA-conjugated FF beads. The graph shows the fluorescence intensity for the detection signal in the presence of PSA at concentrations of 0, 0.020, 0.064, 0.20, 0.64, 2.0, and 6.3 ng/mL at the indicated time. All data are presented as the mean ± the standard deviation (*n* = 4). **d** Schemes for standard immunostaining (top) and magnetically prompted immunostaining of cancer cells using antibody-conjugated FF beads (bottom). **e** The magnetically promoted immunostaining of cancer cells using anti-epidermal growth factor receptor (EGFR)-antibody-conjugated FF beads. Left: Immunostaining of A431 cells (human epidermoid cancer cells, high EGFR expression). Right: Immunostaining of H69 cells (small-cell lung cancer cells, low EGFR expression)

In conventional immunoassays, several processes are time-consuming, such as the antigen–antibody reaction or signal amplification step by an enzymatic reaction. When antibody-labeled fluorescent magnetic beads are collected or concentrated magnetically on an antigen-coated plate, the antigen–antibody reaction will be accelerated. Furthermore, the target antigen can be detected by the direct measurement of the fluorescence of beads. Therefore, we expected FF beads to be suitable for a rapid fluorescence-based immunoassay. Accordingly, using FF beads, we evaluated a sandwich immunoassay system with magnetic collection. Figure 2a illustrates the schemes for conventional sandwich immunoassay systems using enzyme-modified antibodies and a method for introducing magnetic collection using antibody-labeled FF beads. Antigens were immobilized on the antibody-coated plate, and the antibody-labeled FF beads were allowed to react and collected under a magnetic field. Thus, the fluorescence of the FF beads bound to the antigen on the plate can be directly detected [14].

Using this system, we attempted to detect brain natriuretic peptide (BNP) [14], which is a hormone secreted by the heart and a representative disease marker of heart failure [51, 52]. The concentration of BNP in plasma derived from healthy donors is usually less than 20 pg/mL, BNP concentrations of 20–40 pg/mL are associated with heart disease, and concentrations exceeding 100 pg/mL are associated with heart failure [53, 54]. For the detection of BNP using FF beads, we immobilized an anti-BNP antibody on the plate and another anti-BNP antibody on the surface of the FF beads. All operations for the sandwich immunoassay using antibody-coated FF beads were performed by prompt collection under a magnetic field; thereby, the assay, from the addition of the sample to the measurement of fluorescence, could be completed in approximately 5 min. The detection signal of BNP reached a maximum within 1.5 min by the magnetic collection of FF beads, in a dose-dependent manner (Fig. 2b). In contrast, when the sandwich immunoassay was performed without magnetic collection, a stable BNP signal was not detected within a few minutes. Thus, our prompt sandwich immunoassay using FF beads allowed the detection of BNP at a concertation of 2.0 pg/mL with high fidelity within 5 min [14].

We also verified the magnetically promoted sandwich immunoassay system for the detection of a 33–34-kDa glycoprotein enzyme, prostate-specific antigen (PSA) [14], which is a widely used diagnostic biomarker for prostate cancer [55]. Healthy individuals typically have a low concentration of PSA (less than 0.1 ng/mL), and the probability of prostate cancer is about 30% when the PSA concentration is between 4.0 and 10 ng/mL. Therefore, for the clinical examination of prostate cancer, the sensitive detection of PSA at a concentration of 0.1–10 ng/mL is required [56, 57]. Using the magnetically promoted sandwich immunoassay, we detected PSA with 1 min of magnetic collection of FF beads in a dose-dependent manner, and signal saturation was reached within 10 min (Fig. 2c). Furthermore, PSA at a concentration of 0.02 ng/mL was successfully detected within 5 min. These results indicated that the performance of the magnetically promoted sandwich immunoassay system using antibody-coated FF beads was superior to that of conventional immunoassays. Furthermore, with control PSA samples of known concentrations, detection was successful. These results indicate that the magnetic force accelerates specific interactions between antigens and antibodies, and our system could dramatically reduce both the total assay time and the number of steps required for the assay [14].

## Development of a rapid immunostaining method using FF beads

We further applied the magnetically promoted immunoreaction system to the analysis of pathological tissue sections [14]. For tissue diagnosis, immunostaining is frequently used for the detection of pathological regions, such as tumors; however, conventional immunostaining often requires several hours for a definitive diagnosis. We performed an immunostaining assay of cancer cell lines using FF beads immobilized with an antibody against epidermal growth factor receptor (EGFR), which is a cancer marker involved in cancer cell survival and proliferation [58, 59]. As illustrated schematically in Fig, 2d, anti-EGFR-antibody-coated FF beads were added to fixed cancer cell samples. In this step, a permanent magnet was attached beneath the fixed samples to promote the immunoreaction between antigens expressed on the surface of carcinoma cells and antibodies immobilized on FF beads. After the magnet was removed, the samples were washed to remove unbound anti-EGFR-antibody-coated FF beads and were directly observed by fluorescence microscopy. When the immunostaining assay was performed under a magnetic field, high EGFR expression was observed as red fluorescence by FF beads in the epidermoid cancer cell line A341, which is known to express high levels of EGFR (Fig. 2e). This assay was completed within 15 min. In contrast, red fluorescence derived from FF beads was minimal in the lung cancer cell line H69, which is known to express low levels of EGFR. Thus, our rapid immunostaining system using FF beads allows the selective detection of target proteins on cultured cells or tissues, such as pathological sections, with short time requirements.

## Development of a novel method to quantify exosomes using magnetic nanobeads

**Fig. 3:**
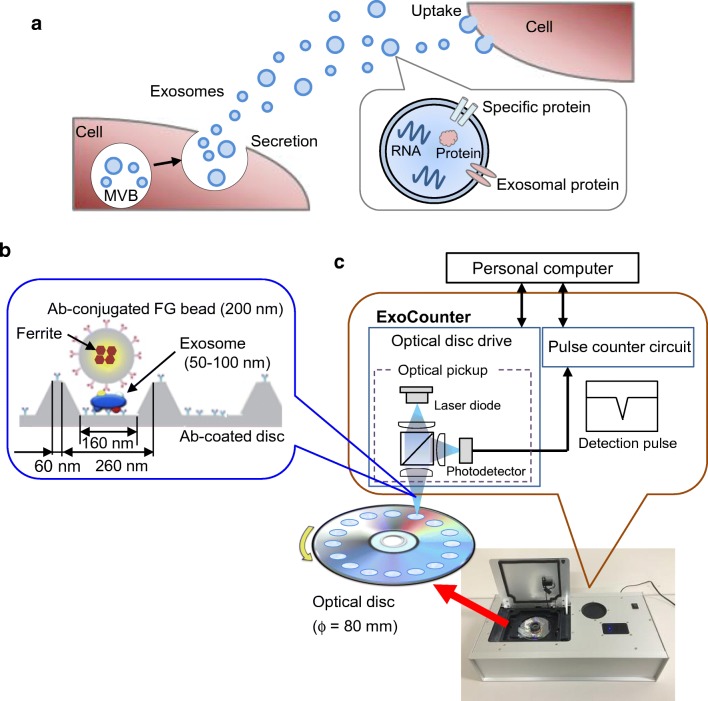

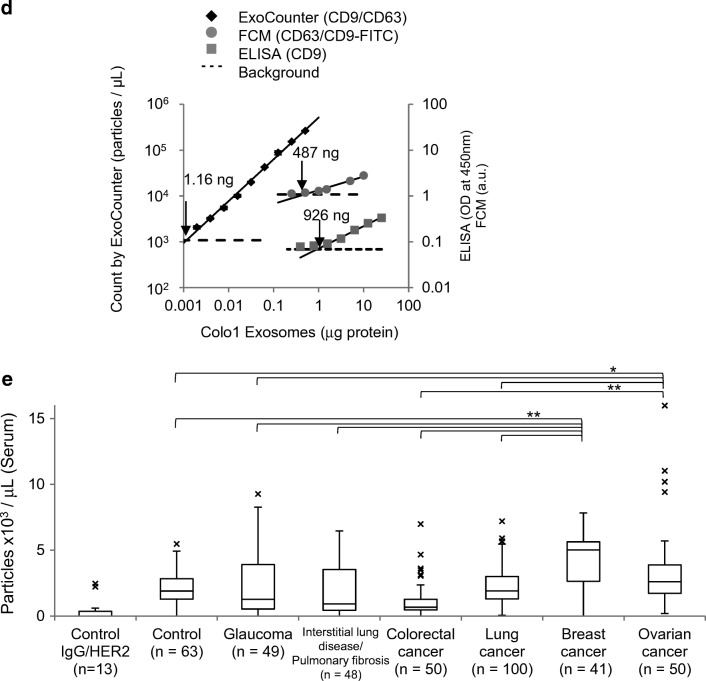
Development of a novel counting system using magnetic nanobeads. Overview of exosomes and the ExoCounter system. **a** Overview of exosomes. When a multivesicular body (MVB) in the cell is fused to the plasma membrane, exosomes are secreted from the cell, delivering genetic material, such as RNAs or proteins, in the membrane to recipient cells. **b** Illustration of exosomes labeled with nanobeads on an optical disc using the ExoCounter system. Each exosome is isolated in the groove of an optical disc coated with a specific antibody (Ab) for exosomes and covered with an antibody-conjugated single magnetic nanobead (FG bead) that contains ferrite particles. The optical disc has periodic grooves of 260 nm (width) at the top and 160 nm at the bottom, which is suitable for the binding of a single exosome (50–150 nm) or FG bead (200 nm). The width of the convex region was 60 nm to prevent the immobilization of exosomes and FG beads outside the groove. **c** Optical disc drive of the ExoCounter system. The captured FG beads on the disc are detected by an optical pickup composed of a laser diode and a photodetector. The detection pulses are transferred to the pulse counter circuit to quantify the number of exosomes. **d** Comparison of Colo1 exosome quantification using the ExoCounter system, enzyme-linked immunosorbent assay (ELISA), and flow cytometry (FCM). **e** Serum samples were incubated on discs coated with anti-CD9 antibody or a control antibody, then treated with FG beads conjugated to an anti-human epidermal growth factor receptor 2 antibody, and analyzed with the ExoCounter system. Serum samples (12.5 μL) from healthy donors or patients with noncancer diseases (glaucoma or interstitial lung disease/pulmonary fibrosis) or cancer (colorectal, lung, breast, or ovarian cancer) were used in the assay. The data are presented in box plots that represent the first quartile (25%), median (50%), and third quartile (75%). The averages for each group are presented below the graph. Data were analyzed statistically by ANOVA with the Tukey–Kramer test. One asterisk *p* < 0.05, two asterisks *p* < 0.01. FITC fluorescein isothiocyanate. (**b**–**e** Reproduced from [13], with permission from the American Association for Clinical Chemistry)

In addition to the development of immunoassays using FF beads, we recently developed a novel system for quantifying the absolute number of exosomes by integrating the magnetic nanobead technology with the optical disc system. Exosomes are cell-secreted membranous vesicles of around 100-nm diameter [3, 60]. When multivesicular bodies in cells fuse to the plasma membrane, the cells release exosomes into the extracellular space [3] (Fig. 3a). Exosomes contain proteins or genetic material such as messenger RNA or microRNA inside a vesicle [61]. Exosomes are present in body fluids, such as blood, urine, saliva, breast milk, semen, ascites fluid, and cerebrospinal fluid, and contribute to intercellular communication by carrying proteins [62] and genetic material through the circulation from parent cells to recipient cells [63]. Through intercellular communication, exosomes play important roles in regulating physiological processes and mediating the systemic dissemination of various types of cancer [64]. On the surface, exosomes ubiquitously express membrane proteins, such as tetraspanin, CD9, CD63, and CD81, called “exosomal proteins.” Furthermore, various specific surface proteins from the parent cell are expressed in exosomes, for instance, CD147 from colorectal cancer cells [65, 66], human epidermal growth factor receptor 2 (HER2) from breast cancer cells [67], and CD91 from lung cancer cells [68], suggesting that exosomes containing specific antigens could be disease biomarkers. Therefore, the detection of exosomes has important practical applications.

Several methods have been developed to detect exosomes, and these can be divided into two general categories: particle-counting methods and labeling-detection methods (Table 1). Nanoparticle tracking analysis (NTA) [69, 70] and tunable resistive pulse sensing (TRPS) [71, 72] are commonly used as particle-counting methods. NTA can be used to determine the number and size of vesicles in solution by analyzing Brownian motion with a laser-illuminating optical microscope [69, 70]. TRPS can be used to analyze the size distribution of particles by detecting electric pulses generated by the change in resistance that occurs when the particles pass through a nanopore in the electrolytic solution [71, 72]. Whereas both NTA and TRPS can analyze the size distribution of exosomes, they cannot distinguish exosomes from other particles, such as protein aggregates or apoptotic bodies. Therefore, the preisolation of exosomes is necessary, for example, by ultracentrifugation [78], size-exclusion chromatography [78], or enrichment with precipitation reagents (e.g., polyethylene glycol) [79]. On the other hand, labeling-detection methods such as ELISA [68, 73] and flow cytometry [62, 74] detect exosomes using specific antibodies against surface antigens on exosomes. In ELISA, exosomes are immobilized on a solid phase and labeled with fluorescent-tag-conjugated or enzyme-conjugated specific antibodies for optical detection to quantify exosomes. In flow cytometry, exosomes are immobilized with antibody-conjugated microbeads and then analyzed by labeling with fluorescent-tag-conjugated antibodies. However, these conventional labeling methods provide only relative quantities of exosomes. Several additional methods have recently been developed [80, 81]. Shao et al. [75] developed a microfluidic device for the detection of labeled exosomes using magnetic nanoparticles. In this method, exosomes immobilized with magnetic nanoparticles are collected in the flow path of a detection chamber, followed by detection by nuclear magnetic resonance using a magnetic coil. This device has sufficient sensitivity to detect 10,000 exosomes. Fang et al. [76] reported a microfluidic chip for detection of exosomes using magnetic nanoparticles. Exosomes were premixed with anti-CD63-antibody-conjugated magnetic nanoparticles and introduced into the microfluidic chip with the primary antibody. The particles were retained in the chamber by a magnet disc, and a fluorescently modified secondary antibody was introduced to label exosomes on the magnetic nanoparticles for detection using an inverted fluorescence microscope. Park et al. [77] developed a device using gold nanoparticles to label exosomes. With use of a thin gold film deposited on nanoholes (220 nm in diameter) coated with an antibody, proteins prepared from exosomes were captured around the nanoholes and labeled with the gold nanoparticles, resulting in a change in the spectrum detected by surface plasmon resonance. Exosomal proteins were quantified by measurement of the spectral shift with a spectrometer. Although these devices quantify the amount of specific proteins expressed in exosomes, the results indicate only the relative quantity of exosomes

**Table 1:** Methods for exosome quantification

Method	Label	Principle	Sensitivity	References
Particle-counting methods				
NTA	No label	Analysis of the Brownian motion of particles by laser imaging	Isolated exosomes 1 × 10^7^/mL	[69, 70]
TRPS	No label	Electrical detection of particles passing through nanopores		[71, 72]
Labeling methods				
ELISA	Fluorescent, chemical luminescent	Enzyme-linked immunosorbent assay	ND [13]	[68, 73]
Flow cytometry with microbeads	Fluorescent	Exosomes captured by microbeads are labeled with fluorescently labeled antibodies for detection by flow cytometry	Serum 14 μL [13]	[62, 74]
nuclear magnetic resonance detection	Magnetic nanoparticle	Exosomes labeled with magnetic nanoparticles are detected by nuclear magnetic resonance	~10,000 exosomes	[75]
Nanoparticle capture by microfluidics	Fluorescent	Exosomes captured by magnetic nanoparticles are labeled with fluorescently labeled antibodies for detection by fluorescence microscopy	Plasma <50 μL	[76]
iNPS	Au nanoparticle	Exosomal proteins are captured around nanopores and labeled with Au nanoparticles to generate surface plasmon resonance for detection by spectroscopy	~10,000 exosomes	[77]
Hybrid method				
ExoCounter	FG beads	Exosomes captured on an optical disc are labeled with FG beads for detection using an optical disc drive by diffraction	Serum 0.39 μL	[13]

*ELISA* enzyme-linked immunosorbent assay, *iNPS* intravesicular nanoplasmonic system, *NTA* nanoparticle tracking analysis, *TRPS* tunable resistive pulse sensing, *ND* not detected

For the accurate detection of the number of exosomes expressing specific marker proteins, we have developed a novel device to quantify specific exosomes with high accuracy by combining optical disc technology with nanobead (FG bead) technology, named “ExoCounter” [13]. In this system, the entire area of optical discs has concentric nanogrooves 260 nm wide at the top and 160 nm wide at the bottom; these are suitable for binding by a single exosome (50–100 nm in diameter) or a single FG bead (200 nm in diameter). A convex region between grooves was designed with a width of 60 nm to prevent the immobilization of exosomes or FG beads outside the groove (Fig. 3b). In addition, FG beads with a diameter of 200 nm are optimal for detection by a laser spot of an optical disc drive (wavelength of 405 nm) and generate significant modulation of the amplitude of the detection signal compared with background noise. With this system, exosomes are captured on antibody-coated optical discs and then labeled with antibody-conjugated FG beads. The number of exosomes can be measured by detection of the FG bead–exosome complexes on the optical disc using an optical disc drive (Fig. 3c). Furthermore, by use of different sets of antibodies, several exosomes expressing specific antigens can be detected. Thus, the ExoCounter system exhibits the advantages of both particle count methods and labeling methods.

To evaluate the properties of the ExoCounter system, we analyzed exosomes isolated from the colorectal cancer cell line Colo1. Exosomes were detected only when exosome-specific antibodies were used, indicating that the ExoCounter system has high selectivity (Fig. 3d). In addition, with anti-CD9-antibody-coated discs and anti-CD63-antibody-conjugated FG beads, exosomes were detected in a dose-dependent manner by the ExoCounter system. Compared with conventional labeling methods, such as ELISA and flow cytometry, the detection sensitivity based on the limit of detection for the ExoCounter system was 800-fold higher than that for ELISA (ExoTest™) and 420-fold higher than that for the flow cytometry system. Furthermore, the linearity, as calculated by the coefficients of determination for the linear regression (*R*^*2*^), for the ExoCounter system was superior to that for ELISA or flow cytometry. The ExoCounter system allows quantification of exosomes derived from clinical samples that include colorectal cancer, breast cancer, or lung cancer cells using FG beads conjugated to antibodies against exosomal or cancer-related proteins. In particular, CD9/HER2 double-positive exosomes were observed in the sera of patients with colorectal cancer and breast cancer cells to a greater extent than in those with other malignancies.

Since it is difficult to directly quantify exosomes in crude samples, such as sera, by conventional methods, a preenrichment procedure is generally necessary for reliable results. We found that the ExoCounter system can detect the exact number of exosomes in human serum without any enrichment procedures. On the basis of this result, we attempted to detect disease-specific exosomes in the sera of patients with and without cancer obtained from the Biobank Japan Project and the sera of healthy donors obtained from the Tohoku University Tohoku Megabank Organization using the ExoCounter system. The numbers of CD9/HER2 double-positive exosomes were significantly increased in the sera of patients with breast cancer or ovarian cancer compared with other samples, suggesting that HER2-expressing exosomes were specifically released from the lesions of breast or ovarian cancer (Fig. 3e). Despite qualitative studies reporting a higher number of HER2-carrying exosomes in the sera of patients with cancer, our results [13] provided the first evidence for a specific increase in the number of HER2-positive exosomes in the sera of patients with cancer based on the accurate quantification of exosomes.

In addition to exosomes secreted in cancer patients, other types of specific exosomes are found in patients with various diseases. For instance, exosomes expressing L1 cell adhesion molecule or neural cell adhesion molecule include disease-associated proteins, such as β-amyloid and α-synuclein, and may be related to neurodegenerative diseases, such as Alzheimer’s disease and Parkinson’s disease [82, 83]. In addition, other researchers reported unique surface proteins of exosomes related to autoimmune diseases [84] or depression [85]. Many studies have also suggested the importance of specific exosomes for physiological analyses or therapeutic applications [86, 87]. We have recently developed an automated pathological diagnosis system for cancer tissues based on an enforced-learning platform [88]. The combination of imaging technology for on-tissue cancer diagnosis and the ExoCounter system for quantitative exosome analyses may allow comprehensive and objective approaches for the precise diagnosis of malignancies. The ExoCounter system is commercially available from JVCKENWOOD Corporation (http://healthcare.jvc.com/exosome/).

## Conclusion

In conclusion, by applying our magnetic nanobead technology, we successfully developed a rapid immunoassay system and a quantification system for the absolute number of exosomes, beginning with affinity purification. The development of these systems was possible because of the excellent properties of the beads, such as their strong paramagnetism, physical stability, low nonspecific adsorption, and uniform dispersibility with a nanosized diameter. Further analyses are expected to lead to the development of novel diagnostic systems based on our high-performance magnetic nanobeads.
